# Supplementary material for: Global trends in antimicrobial use in food-producing animals: 2020 to 2030
Source: PLOS Glob Public Health. 2023 Feb 1;3(2):e0001305. doi: 10.1371/journal.pgph.0001305 (PMC10021213; doi:10.1371/journal.pgph.0001305)
Supplement: S1 Protocol — (DOCX) [file pgph.0001305.s001.docx]

**S1 Protocol:** *Extrapolation of Antimicrobial Usage*

We used a five-step statistical procedure adapted from Van Boeckel et al, 2015 [2], to extrapolate antimicrobial usage (AMU) for 13 classes of antimicrobials from countries reporting sales, consumption, usage or imports (henceforth referred to as AMU) (n=42), to all other countries (n=187). The objective of this procedure was to calculate AMU for each class of antimicrobials (c=13) in each country (i=229) for each group livestock species (k=4) and in each production system (s = extensive *or* intensive). AMU was calculated in tonnes, and AMU intensity in mg per population correction units (PCU) - mg/PCU.

**Step 1: Usage Intensity.** We used a multivariate regression model using data from the 42 countries reporting national-level AMU data to establish a statistical relationship between the overall AMU and the PCU of chicken and pigs raised in intensive systems (there was no significant relationship for cattle and as such was not included). This relationship was subsequently used to predict overall AMU intensity in intensive systems (mg/PCU_int_) in all other countries.

**Step 2: Tetracyclines use vs Consumption Intensity.** We used a univariate regression model including all reporting countries to establish a relationship between the AMU intensity in intensive systems (mg/PCU_int_) and the percentage tetracycline π_TET_ of the total AMU (S1 Fig). This relationship was subsequently used to predict the proportion of tetracyclines used in each country according to its AMU intensity across all antibiotic classes (Step 1). This was done to reflect the fact that tetracyclines are proportionally overused in high-consuming countries (which were the vast majority of reporting countries). The proportions of non-tetracycline compounds for each modelled country were derived from the mean proportions of each of those compounds across all reporting countries (as no significant relationships between AMU intensity and overuse of compounds other than tetracyclines were found). All proportions were adjusted to sum up to one.

**Step 3: Species-specific consumption.** We used species-specific estimates of AMU from 19 countries (with data since 2010) to compute average species-specific relative proportions for different compounds (π_k_,_c_). For countries where AMU by species was provided by class of compound (SCB) at the national level, these values were used directly to compute coefficients of AMU for intensive systems. For countries where species AMU levels were aggregated across compounds (SB), this overall volume was disaggregated into 13 α_c,i,k,s_ coefficients of AMU intensity, scaled according to the median proportions π_c,k_ computed in SCB countries. Finally, in every country the value of the species-specific AMU for tetracycline (α_c,TET,s_) was adjusted (Step 2) and the AMU for other compounds was adjusted accordingly. Finally, in the countries where species-specific estimates of the national AMU were available, these values were used but with these α_c,i,k,s_ coefficients scaled to match the values reported by those countries.

**Step 4: Extensive vs intensive production systems.** In line with Van Boeckel et al, 2015 [2], we assumed that intensive systems consume on average four times the amount when compared to extensive systems, per kilogram of livestock produced. Finally, in each country, estimates of the global AMU by species and production system were obtained by multiplying the coefficients of AMU by the number of PCU in each corresponding livestock system. Confidence intervals on the total AMU were calculated as 1.96 times the standard deviation associated with the coefficients of AMU per species (α_c,I,k,s_, step 3). Negative predictions for the lower bound were truncated to zero. The (unadjusted, see step 5) AMU and its associated confidence interval (95% C.I.) was estimated in each country I such as:

$${AMU}_{Pred,i}= \sum_{c}^{13} \sum_{k}^{4} \sum_{s}^{2} {(\alpha}_{c,k,s}\pm1.96 \cdot sd(\alpha_{c,k,s}))\cdot{PCU}_{2020,c,k,s}$$

where *sd*(α_c,I,k,s_) is the standard deviation on the estimated coefficient from step 3.

**Step 5: Matching WOAH’s regional estimates.** The World Organization for Animal Health (WOAH) conducts yearly surveys that enable countries to confidentially report veterinary antimicrobial usage, sales, consumption, and imports (as proxy for usage), and these are subsequently aggregated regionally and released in a public report [8]. This report was last updated with data from year 2018, and combines sales data from 109 countries in five regions: Europe, Africa, Americas, Middle East, Asia/Oceania. In the latest report, just three countries contributed data in the Middle East region. Rather than rely on data from only a quarter of the countries in the region (3 of 12), we calculated the regional consumption intensity in the Middle East as the average between that of Africa and Asia.

WOAH report two antimicrobial quantities in tonnes (kg) – the first is the absolute value reported by countries, and the second is adjusted by a coverage estimate (%) requested by WOAH. We used the latter value in this analysis to better represent full national coverage of AMU.

WOAH report total AMU as a combination of terrestrial and aquatic animals. However, in this study we estimate antimicrobial usage in terrestrial animals only. To obtain AMU totals per region for terrestrial animals only, we assume average AMU intensity is comparable between aquatic and terrestrial animals, and multiply the total AMU reported by the proportion of terrestrial animals’ biomass of the total animal biomass.

While the WOAH report does not disclose which countries participated (RC) or did not participate (NRC) in their annual surveys, it reports an estimate of the animal biomass per region (PCU_REG,WOAH_), as well as the percentage of this biomass that is associated with RC (%PCU_REG_,_WOAH_).

We used the information collected by WOAH to scale our predictions of AMU by country such that it matches the average AMU per unit of biomass (mg/PCU) in each region, as well as the national reports of AMU when these had been published independently from the WOAH annual survey on antimicrobial use (Table S1). We proceeded in 5 sub-steps:

**Step 5.1.** We calculated the absolute regional AMU for all countries in each region from the WOAH report in 2018 under the assumption that the average usage intensity [mg/PCU_WOAH,REG_] calculated from the reporting countries (RC) could be extrapolated to the entire region, including non-reporting countries (NRC) based on regional biomass covered in the WOAH annual survey. Such as,

AMU2018_REG_ = [mg/PCU_WOAH,REG_] x (PCU_WOAH,REG_ / PropPCU_REG_,_WOAH_).

**Step 5.2** We calculate for each region, the AMU intensity for 2018 using the PCU defined by Van Boeckel et al, 2015 [2]. Such that,

[mg/PCU_REG_]_2018_ = AMU2018_REG_ / PCU_REG_

**Step 5.3** We calculated, R_2020/2018_, the global ratio of usage intensity between 2020 and 2018 using published national reports. The calculation only included countries that reported for both 2018 and 2020.

**Step 5.4** We calculated the regional AMU intensity for 2020 by multiplying the AMU intensity for 2018 with R_2020/2018._

[mg/PCU_REG_]_2020_ = [mg/PCU_REG_]_2018_ x R_2020/2018._

**Step 5.5** We calculated a scaling factor φ_,REG_ for each coefficient of AMU $\alpha_{c,i,k,s}$. For NRC, AMU coefficients ($\alpha_{c,i,k,s,NRC}$) were scaled for each class by a factor φ_,REG_ such that it satisfied the average regional mean reported by WOAH after imputing national reported national usage in that region such that:

φ_,REG_ = (([mg/PCU_REG_]_2020,REG_ x PCU_2020_,_REG_) - AMU_2020,REG,Obs_) / (AMU_2020,Pred,REG_).

For RC, consumption coefficients ($\alpha_{c,i,k,s,RC}$) were scaled for each class of compound, and species such that the amount of antimicrobial predicted in a country matched the amount antimicrobial reported by each country.

*Obs = observation and Pred = prediction.*
