# Supplementary figures and images for: Global trends in antimicrobial use in food-producing animals: 2020 to 2030
Source: PLOS Glob Public Health. 2023 Feb 1;3(2):e0001305. doi: 10.1371/journal.pgph.0001305 (PMC10021213; doi:10.1371/journal.pgph.0001305)

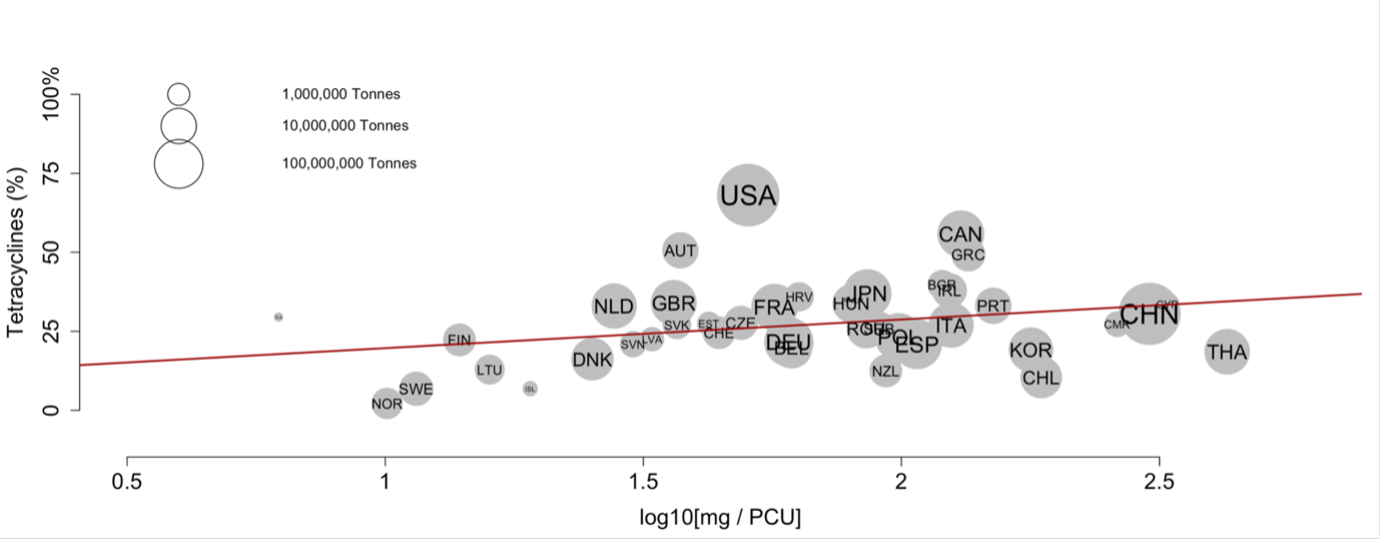

Supplement: S1 Fig — Circles are proportional to the log10 of the PCU in each country. (TIFF) [file pgph.0001305.s002.tiff]

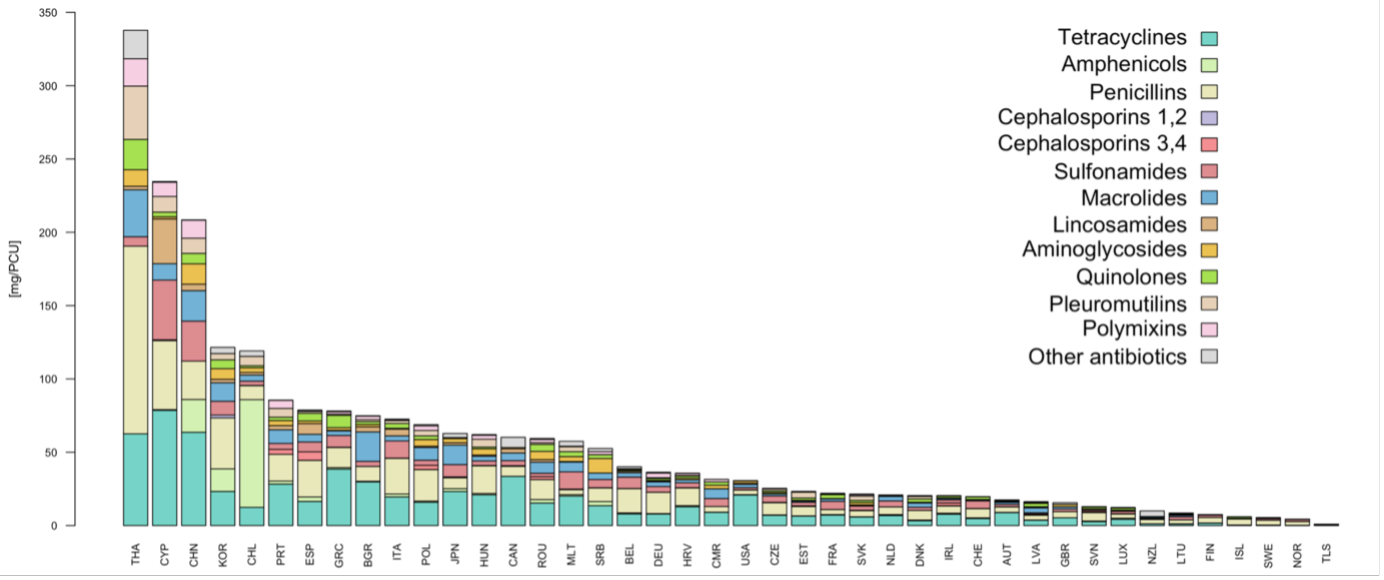

Supplement: S2 Fig — All countries are referred to by their ISO3 alpha-3 code, found: https://www.iso.org/obp/ui/#search. (TIFF) [file pgph.0001305.s003.tiff]

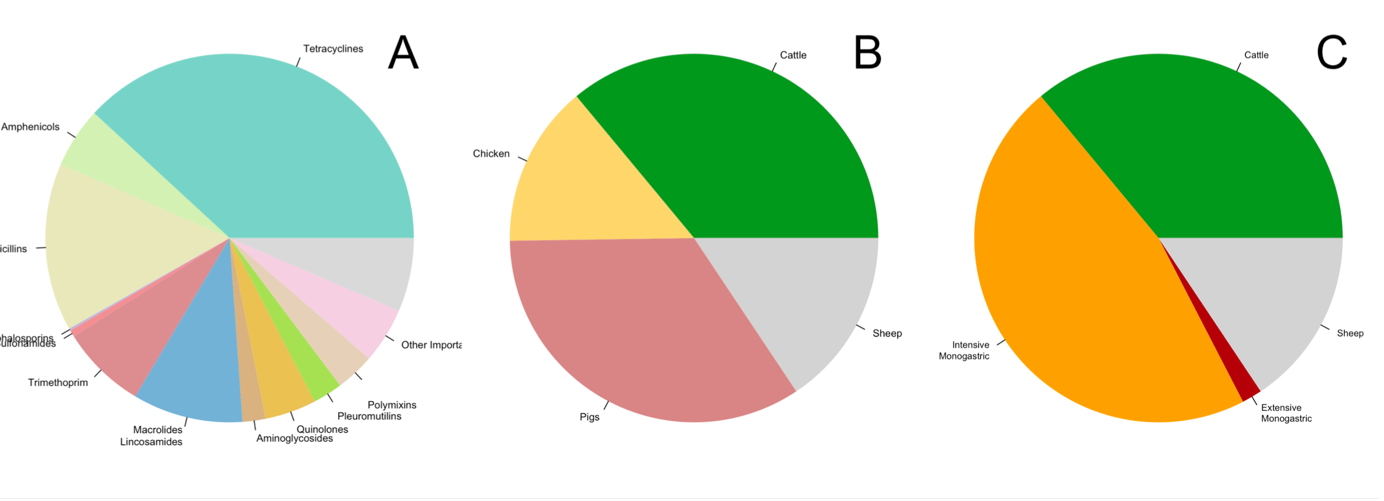

Supplement: S3 Fig — Proportion of veterinary antimicrobial usage in 2020 by (A) antimicrobial class, (B) animal species, and (C) type of animal. (TIFF) [file pgph.0001305.s004.tiff]

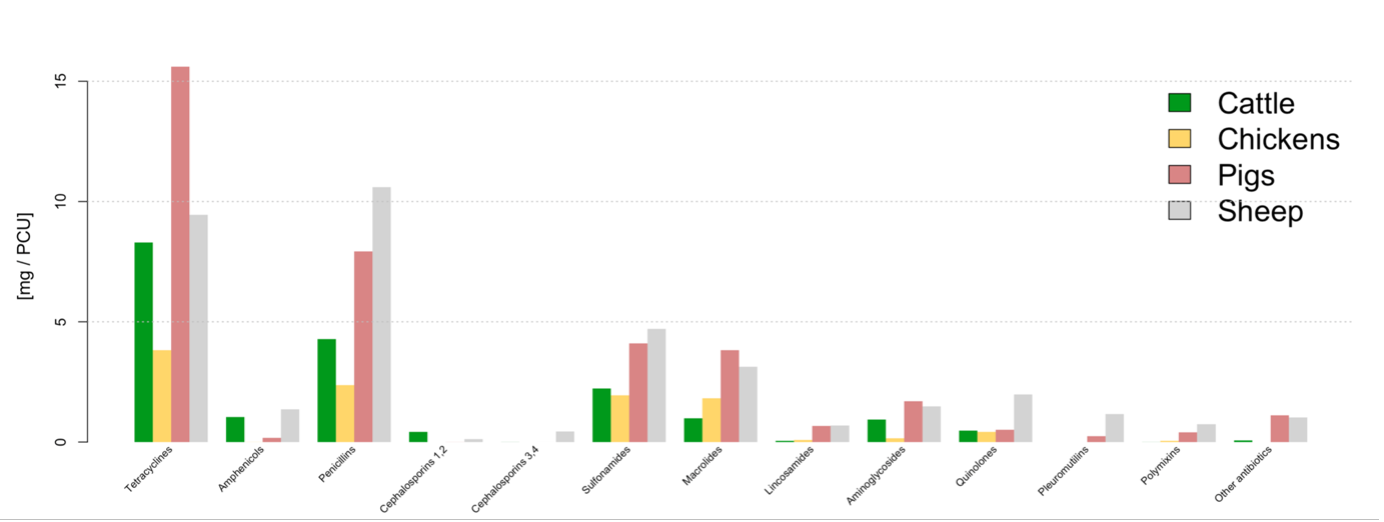

Supplement: S4 Fig — (TIFF) [file pgph.0001305.s005.tiff]
