# Supplementary material for: Global trends in antimicrobial use in food-producing animals: 2020 to 2030
Source: PLOS Glob Public Health. 2023 Feb 1;3(2):e0001305. doi: 10.1371/journal.pgph.0001305 (PMC10021213; doi:10.1371/journal.pgph.0001305)
Supplement: Attachment — Submitted filename: PGPH_response_to_reviewers_final.docx [file pgph.0001305.s011.docx]

**Global trends in antimicrobial use in food-producing animals: 2020 to 2030**

**PGPH-D-22-01542**

Thank you very much to all reviewers and editor for their constructive feedback. Below we have outlined -in red- the changes made to our manuscript to incorporate the feedback of the reviewers.

Journal requirements

1. Review reference list Done
2. Complete “Competing Interests’ statement”, including COIs were relevant.

Added a competing interest statement on line 25*.*

3. Insert Ethics Statement Added on line 114 that *“no ethical approval was required for this study. All data were retrieved from open access”.*
4. Provide separate figure files Done

Reviewer #1:

Here the authors present the Global trends in antimicrobial use in food-producing animals from 2020 to 2030. Antimicrobial resistance is a major public health and clinical concern globally, and antimicrobial use in food animals have been implicated as one of its drivers. The concept of the study is timely, as the objectives provide additional information. The concept, study design, and analysis of the study were very good, including the presentation of results and discussion.

Some minor comments to improve the quality of the manuscript.

Line 28: Add "in" after "helped". Done

Line 36: Change "animals" to "animal" Done

Line 129: Delete "cephalosporins" as it appears twice. Done

Line 130: Correct spelling of "Polymixins" to " Polymyxins". Add "and" after "Polymyxins" and "s" to "other". Delete ")" after "other". Done

Line 132: Write "5" in words. Done

Line 136: Complete sentence "for pooled...across animal species" what? Not clear Done

Line 145: Correct "on-off" to "one off". Done

Reviewer #2:

An interesting manuscript on the Global trends in antimicrobial use in food-producing animals: 2020 to 2030. In this study, the authors estimated antimicrobial use (tonnes) calibrated to match continental-level reports of antimicrobial use intensity (milligrams per kilogram of animal) from the World Organization of Animal Health, as well as country-level reports of antimicrobial use from countries. Very good efforts.

The manuscript is well articulated but needs minor review.

L101: Insert ‘of’ after ‘…. number’. Done

L250: Insert ‘comma ( , )’after ‘… tonnes’ Done

L251: Insert ‘comma ( , )’after ‘… tonnes’ Done

Reviewer #3:

This manuscript is technically sound and does have verifiable data to support findings and conclusions, but not without minor errors that will require revision.

Information on lines 97 through 103 is better suited for materials and methods, while emphasis should be placed on the paper's stated goal.

This has now been removed (due to the duplication with the materials and methods). The final two sentences of the introduction now emphasize the paper’s main aims, as follows:

*“In this study, statistical models [2] were used in combination with country reports of veterinary AMU, regional totals of AMU from WOAH, and on-farm AMU surveys, to estimate AMU in food-producing animals for 229 countries/territories in 2020. Global AMU was estimated to 2030, and AMU was mapped at 10x10km resolution to identify global hotspots of high AMU.”*

Line 107 should be restructured for clarity. The report of "veterinary usage" stated in the sentence was unclear.

This has been re-written for clarity, as follows:

*“An online search was conducted from three sources of data 1) government reports on veterinary antimicrobials (predominantly sales) at country-level, collected through established surveillance systems, 2) scientific articles reporting estimates of veterinary AMU at country-level (predominantly imports), and 3) scientific articles reporting on-farm AMU from surveys within countries.”*

Line 114 through 124 should be restructured to include the year range for literature search, which were not stated in the manuscript or the supporting document.

PubMed was searched for any article published between 01 January 2010 and 01 June 2022. Only national-level data published after 2017 (the baseline for previous iteration of this analysis – Tiseo et al, 2021) were included in this analysis. For species-specific estimates, data from any article published after 2010 were retained. The paragraph has been updated as follows:

*“The literature search was conducted in PubMed using the following search term: Veterinary (antibiotic OR antimicrobial) (use OR usage OR consumption OR sales) AND (antibiotic OR antimicrobial) (use OR usage OR consumption OR sales) AND (animals OR food animals OR livestock OR swine* OR pig* OR poultry* OR chicken* OR cattle* OR dairy* OR beef* OR sheep*). Between 01 January 2010 and 01 June 2022, 4,217 articles were identified, and all had their titles and abstracts screened. 126 articles underwent a full text read. 10 articles contained national data on antimicrobial sales, import, or consumption. Of these, four articles came from countries that did not have any national surveillance system; however, only half (n=2) contained data post-2017 and only those were retained (Cameroon, Timor-Leste) (Table S2). An additional four articles were identified to contain sub-national species-level survey data only (Brazil, Fiji, Morocco, and Pakistan) (Table S3).”*

The authors need to provide information on how bias/risk assessments were carried out in the materials and methods

We acknowledge bias towards the potential over representation of high-income countries in the limitation section on line 334-335:

*“…the number of countries for which country-level AMU reports could be identified between 2018 and 2020 was limited (n=42), the majority of which came from high-income countries”*.

Regarding the inevitable uncertainty results from the modelling, we calculated uncertainty intervals around our estimates. The details on the calculation used can be found in the Supplementary Materials under Step 4 of Protocol S1.

*“In line with Van Boeckel et al, 2015 [2], we assumed that intensive systems consume on average four times the amount when compared to extensive systems, per kilogram of livestock produced. Finally, in each country, estimates of the global AMU by species and production system were obtained by multiplying the coefficients of AMU by the number of PCU in each corresponding livestock system. Confidence intervals on the total AMU were calculated as 1.96 times the standard deviation associated with the coefficients of AMU per species (αc,i,k,s, step 3). Negative predictions for the lower bound were truncated to zero. The (unadjusted, see step 5) AMU and its associated confidence interval (95% C.I.) was estimated in each country i, where sd(αc,i,k,s) is the standard deviation on the estimated coefficient from step 3”.*

$${AMU}_{Pred,i}= \sum_{c}^{13} \sum_{k}^{4} \sum_{s}^{2} {(\alpha}_{c,k,s}\pm1.96 \cdot sd(\alpha_{c,k,s}))\cdot{PCU}_{2020,c,k,s}$$

The authors did not state clearly or highlight their conclusions and recommendations that could negate any possible negative projections made.

We thank the reviewer for this suggestion. We have added a sentence on lines 354-356 to the discussion to state that our modelling approaches were conducted under the BAU scenario and acknowledge that countries may take actions that will result in curbing AMU prior to 2030.

*“Thirdly, projections within this study were conducted under a BAU scenario. As such, where countries take actions to curb AMU in the coming years, these projections with be likely over-estimates of AMU by 2030”*

In conclusion, authors are advised to subject this write-up to the assessment of a native English speaker to take care of minor typographical and grammar errors.

The full manuscript has been reviewed by a native English speaker to identify any remaining typographical or grammatical errors.
